# Three new species of *Conidiobolus* sensu stricto from plant debris in eastern China

**DOI:** 10.3897/mycokeys.73.56905

**Published:** 2020-10-08

**Authors:** Yong Nie, Yue Cai, Yang Gao, De-Shui Yu, Zi-Min Wang, Xiao-Yong Liu, Bo Huang

**Affiliations:** 1 Anhui Provincial Key Laboratory for Microbial Pest Control, Anhui Agricultural University, Hefei 230036, China; 2 School of Civil Engineering and Architecture, Anhui University of Technology, Ma’anshan 243002, China; 3 Department of Biological and Environmental Engineering, Hefei University, Hefei 230601, China; 4 Bioengineering and Technological Research Centre for Edible and Medicinal Fungi, Jiangxi Agricultural University, Nanchang, 330045, China; 5 State Key Laboratory of Mycology, Institute of Microbiology, Chinese Academy of Sciences, Beijing 100101, China

**Keywords:** basal fungi, *
Entomophthorales
*, taxonomy, molecular phylogenetics, new species

## Abstract

The genus *Conidiobolus* Bref. is widely distributed and the *Conidiobolus* sensu lato contained three other genera, *Capillidium*, *Microconidiobolus* and *Neoconidiobolus*. A molecular phylogeny based on the nuclear large subunit of rDNA (nucLSU), the mitochondrial small subunit of rDNA (mtSSU) and the translation elongation factor 1-alpha gene (TEF1) revealed three novel species within the clade of *Conidiobolus* s.s., i.e. *C. bifurcatus***sp. nov.**, *C. taihushanensis***sp. nov.** and *C. variabilis***sp. nov.** These three species were isolated from plant debris in eastern China. Morphologically, *C. bifurcatus***sp. nov.** is characterised by its secondary conidiophores often branched at the tip to form two short stipes each bearing a secondary conidium. *C. taihushanensis***sp. nov.** is different from the others in its straight apical mycelia and the production of 2–5 conidia. *C. variabilis***sp. nov.** is distinctive because of its various shapes of primary conidia. All these three new taxa are illustrated herein with an update key to the species of the genus *Conidiobolus* s.s.

## Introduction

The genus *Conidiobolus* Bref. (Ancylistaceae) was established to accommodate the type *C. utriculosus* Bref. and a second species *C. minor* Bref. (Brefeld 1884). This genus was characterised by simple conidiophores, globose to pyriform conidia and resting spores formed in the axis of the hypha (mostly as zygospores) (Humber 1997). Until 1968, a total of 41 species occurring saprotrophically in soil and plant debris had been assigned to this genus (Martin 1925, Couch 1939, Drechsler 1952, 1953a, b, 1954, 1955a, b, c, 1956, 1957a, b, c, 1960, 1961, 1962, 1965, Srinivasan and Thirumalachar 1961, 1962a, b, 1965, 1967, 1968a, b). In a review of these taxa with the numerical technique, 27 definitive species were recognised (King 1976a, b, 1977). On the basis of the shape of secondary conidia, Ben-Ze’ev and Kenneth (1982) classified the genus *Conidiobolus* into three subgenera, including *Capillidium* Ben-Ze’ev & Kenneth, *Conidiobolus* Brefeld and *Delacroixia* Tyrrell & Macleod. Until 2018, no remarkable taxonomic treatments had been made for this genus, although additional species were reported continuously (Bałazy et al. 1987, Waters and Callaghan 1989, Bałazy 1993, Tosi et al. 2004, Huang et al. 2007, Waingankar et al. 2008, Nie et al. 2012, 2016, 2017, 2018). Meanwhile, higher-rank molecular phylogenetic studies on entomophthoroid fungi suggested *Conidiobolus* to be polyphyletic (Jensen et al. 1998, Gryganskyi et al. 2013, Nie et al. 2020). Consequently, the three genera *Capillidium*, *Microconidiobolus* and *Neoconidiobolus* were separated from *Conidiobolus* sensu lato and *Conidiobolus* sensu stricto was characterised by microspores arising from conidia (Nie et al. 2020).

During the past decade, Bo Huang’s research group have carried out a comprehensive study on the taxonomy of *Conidiobolus* sensu lato in China and proposed five new species, five Chinese new records and 23 new combinations (Wang et al. 2010a, b, Nie et al. 2012, 2016, 2017, 2018, 2020, Chen and Huang 2018). Recent collections by this research group in eastern China resulted in the discovery of three unique species within the *Conidiobolus* sensu stricto lineage, which are described and illustrated herein with a multi-locus molecular phylogeny on the nuclear large subunit of rDNA (nucLSU), the mitochondrial small subunit of rDNA (mtSSU) and the translation elongation factor 1-alpha gene (TEF1).

## Materials and methods

### Isolates and morphology

Plant debris was collected from Taihushan and Jilongshan National Forest Parks, Anhui Province, China and Laoshan National Forest Park, Jiangsu Province, China. Isolations were carried out using the canopy-plating approach (Drechsler 1952, King 1976a). A Petri dish with potato dextrose agar (PDA; potato 200 g, dextrose 20 g, agar 20 g, H_2_O 1000 ml) was inverted over the plant debris and incubated at 21 °C for daily examining for one week. When entomophthoroid fungi on the PDA canopy were detected, they were quickly transferred to new PDA and 2% water agar (agar 20 g, H_2_O 1000 ml) plates for purification and description. Morphological features were measured with an Olympus BX51 research microscope for 35 primary conidia and conidiophores each and photographed by an Olympus DP25 microscope-camera system. The descriptions were made with the method of King (1976a). Cultures were deposited in the Research Center for Entomogenous Fungi of Anhui Agricultural University, Anhui Province, China (RCEF) and the China General Microbiological Culture Collection Center, Beijing, China (CGMCC). Dried cultures were deposited in the Herbarium Mycologicum Academiae Sinicae, Beijing, China (HMAS). In order to infer the phylogeny of the genus *Conidiobolus* s.s., a total of 21 ex-types of species in *Conidiobolus* s.l., serving as outgroup, were obtained from the American Type Culture Collection, Manassas, USA (ATCC).

### DNA extraction, PCR amplification and sequencing

Fungal biomass was collected from the plate surface and ground in liquid nitrogen with a pestle and mortar. Genomic DNA was extracted using the CTAB method (Watanabe et al. 2010). The extracted DNA was stored in 100 μl TE buffer (10 mM Tris-HCl, 1 mM EDTA, pH 8.0) at -20 °C. Universal primer pairs LR0R (5'-ACC CGC TGA ACT TAA GC-3') and LR5 (5'-TCC TGA GGG AAA CTT CG-3') (Vilgalys and Hester 1990), mtSSU1 (5'-GCW GCA GTG RGG AAT NTT GGR CAA T-3') and mtSSU2R (5'-GTR GAC TAM TSR GGT ATC TAA TC-3') (Zoller et al. 1999) and EF983 (5'-GCY CCY GGH CAY CGT GAY TTY AT-3') and EF1aZ-1R (5'-ACA TCW CCG ACA CCC TTG ATC TTG -3') (Nie et al. 2012) were used for the amplification of the partial region of nucLSU, mtSSU and TEF1, respectively. The PCR reactions followed those in Liu et al. (2005) and Nie et al. (2012, 2020). A 50 μl mixture contained 200 μM dNTPs each, 1 × Mg-free buffer, 2.5 mM MgCl_2_, 0.5 μM primers each, 50 ng genomic DNA and 2 U Taq polymerase (Super Pfx DNA Polymerase, Cowinbioscience Co. Ltd., Shanghai, China). The programme consisted of an initial denaturation at 100 °C for 5 min without Taq polymerase, an extra denaturation at 95 °C for 5 min after the Taq polymerase was added, then 34 cycles of 94 °C for 1 min plus 55/54/57 °C (nucLSU / mtSSU / TEF1) for 2 min plus 72 °C for 2 min and a final extension at 72 °C for 10 min. The amplification products were sequenced by Shanghai GeneCore BioTechnologies Co. Ltd. (Shanghai, China), with the same primers as used in relative PCR reactions. All sequences were assembled with BioEdit (Hall 1999) and deposited at GenBank (Table 1).

**Table 1.** The taxa used in phylogenetic analyses.

**Species**	Strains*	**GenBank accession numbers**			**References**
		**nucLSU**	**EF-1α**	**mtSSU**	
*Capillidium adiaeretum*	CGMCC 3.15888	MN061284	MN061481	MN061287	Nie et al. 2020
*Ca. lobatum*	ATCC 18153 (T)	JF816218	JF816233	MK301187	Nie et al. 2012, 2020
***Conidiobolus bifurcatus* sp. nov.**	**CGMCC 3.15889 (T)**	**MN061285**	**MN061482**	**MN061288**	This article
*C. brefeldianus*	ARSEF 452 (T)	EF392382	–	EF392495	Genbank
*C. chlamydosporus*	ATCC 12242 (T)	JF816212	JF816234	MK301178	Nie et al. 2012, 2020
*C. coronatus*	NRRL 28638	AY546691	DQ275337	–	Lutzoni et al. 2004
*C. coronatus*	RCEF 4518	JN131537	JN131543	–	Nie et al. 2016, 2018
*C. dabieshanensis*	CGMCC 3.15763 (T)	KY398125	KY402206	MK301180	Nie et al. 2017, 2020
*C. firmipilleus*	ARSEF 6384	JX242592	–	JX242632	Gryganskyi et al. 2012
*C. gonimodes*	ATCC 14445 (T)	JF816221	JF816226	MK301182	Nie et al. 2012, 2020
*C. humicolus*	ATCC 28849 (T)	JF816220	JF816231	MK301184	Nie et al. 2012, 2020
*C. incongruus*	NRRL 28636	AF113457	–	–	Voigt et al. 1999
*C. iuxtagenitus*	ARSEF 6378 (T)	KC788410	–	–	Gryganskyi et al. 2013
*C. khandalensis*	ATCC 15162 (T)	KX686994	KY402204	MK301185	Nie et al. 2012, 2020
*C. lamprauges*	ARSEF 2338	DQ364206	–	DQ364226	Genbank
*C. lichenicolus*	ATCC 16200 (T)	JF816216	JF816232	MK301186	Nie et al. 2012, 2020
*C. macrosporus*	ATCC 16578 (T)	KY398124	KY402209	MK301188	Nie et al. 2017, 2020
*C. megalotocus*	ATCC 28854 (T)	MF616383	MF616385	MK301189	Nie et al. 2018, 2020
*C. mycophagus*	ATCC 16201 (T)	JX946694	JX946698	MK301190	Nie et al. 2018, 2020
*C. mycophilus*	ATCC 16199 (T)	KX686995	KY402205	MK301191	Nie et al. 2016, 2020
*C. parvus*	ATCC 14634 (T)	KX752051	KY402207	MK301192	Nie et al. 2016, 2020
*C. polyspermus*	ATCC 14444 (T)	MF616382	MF616384	MK301193	Nie et al. 2018, 2020
*C. polytocus*	ATCC 12244 (T)	JF816213	JF816227	MK301194	Nie et al. 2012, 2020
***C. taihushanensis* sp. nov.**	**CGMCC 3.16016 (T)**	**MT250086**	**MT274290**	**MT250088**	This article
***C. variabilis* sp. nov.**	**CGMCC 3.16015 (T)**	**MT250085**	**MT274289**	**MT250087**	This article
*Microconidiobolus nodosus*	ATCC 16577 (T)	JF816217	JF816235	MK333388	Nie et al. 2012, 2020
*M. terrestris*	ATCC 16198 (T)	KX752050	KY402208	MK301199	Nie et al. 2016, 2020
*Neoconidiobolus stromoideus*	ATCC 15430 (T)	JF816219	JF816229	MK301198	Nie et al. 2012, 2020
*N. thromboides*	ATCC 12587 (T)	JF816214	JF816230	MK301200	Nie et al. 2012, 2020

*ARSEF, ARS Entomopathogenic Fungus Collection (Ithaca, U.S.A.). ATCC, American Type Culture Collection (Manassas, U.S.A). CGMCC, China General Microbiological Culture Collection Center (Beijing, China). NRRL, ARS Culture Collection (Peoria, U.S.A). RCEF, Research Center for Entomogenous Fungi (Hefei, China). T = ex-type.

### Phylogenetic analyses

In addition to the sequences obtained in this paper, nucLSU, mtSSU and TEF1 sequences of 20 strains in *Conidiobolus* sensu stricto were downloaded from GenBank. Three genera *Capillidium*, *Microconidiobolus* and *Neoconidiobolus*, each represented by two species, were selected as outgroups. The nucLSU, mtSSU and TEF1 sequences were aligned with Clustal X (Thompson et al. 1997) and deposited at TreeBase (submission ID 26063). Phylogenetic analyses with Bayesian Inference (BI), Maximum Parsimony (MP) and Maximum Likelihood (ML) were carried out according to Nie et al. (2018, 2020). BI phylogeny was estimated using MrBayes 3.1.2 (Ronquist and Huelsenbeck 2003). The best-fit model selected with the Akaike Information Criterion (AIC) in Modeltest 3.7 (Posada and Crandall 1998) was used to evaluate Posterior Probabilities (PP) and the critical value for the topological convergence diagnostic was set to 0.01 of the average standard deviation of split frequencies. Four Markov chains ran simultaneously from random starting trees for 0.5 million generations and trees were sampled every 100^th^ generation. MP analyses were performed using a heuristic search with PAUP* 4.0b10 (Swofford 2002). All characters were weighted and gaps were treated as missing data. Tree bisection-reconnection (TBR) was set as the branch swapping algorithm. Branch robustness was estimated with bootstrapping 1,000 replicates (Felsenstein 1985). ML analyses were performed with the RAxML (Stamatakis 2006), implemented in raxmlGUI 1.5b1 (Silvestro and Michalak 2012). Branch reliabilities were determined by 1,000 ML rapid bootstrap replicates with the GTRGAMMA substitution model. Phylogenetic trees were checked and modified in FigTree 1.4 (Rambaut 2012).

## Results

### Phylogenetic analyses

**Figure 1.**
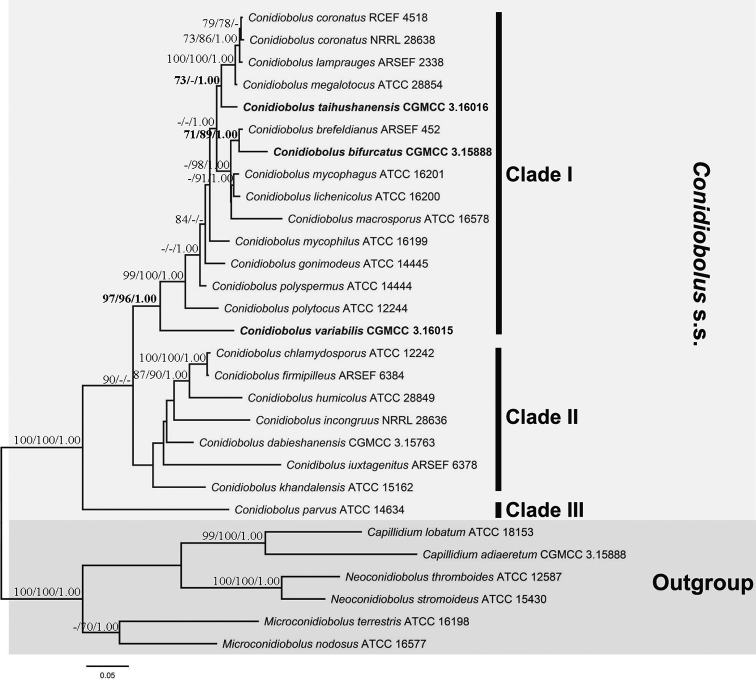
Phylogenetic tree of *Conidiobolus* s.s. reconstructed by maximum likelihood analyses of nucLSU, mtSSU and TEF1 sequences, with six *Conidiobolus* s.l. species as outgroups. Three new species of *Conidiobolus* are shown in bold. Maximum parsimony bootstrap values (≥ 70%) / Maximum likelihood bootstrap values (≥ 70%) / Bayesian posterior probabilities (≥ 0.95) of each clade are indicated along branches. Scale bar indicates substitutions per site.

The combined nucLSU+TEF1+mtSSU dataset was composed of 29 taxa representing 27 species and 1949 characters including 986 constant, 276 parsimony-uninformative and 687 parsimony-informative. The most parsimonious tree was generated with a tree length (TL) of 2716 steps, a consistency index (CI) of 0.5497, a homoplasy index (HI) of 0.4503, a retention index (RI) of 0.6191 and a rescaled consistency index (RC) of 0.3403. The best model applied in the BI analysis was GTR+I+G. The final average standard deviation of split frequencies was 0.0086 and the final likelihood value was -14423. The three phylograms resulted in similar topologies and the ML tree was presented along with MP/ML bootstrap and BI posterior probability values at relative branches (Fig. 1).

Three clades can be seen to form for the *Conidiobolus* s.s. The three species, described here, were located in clade I.

### Taxonomy

#### Conidiobolus bifurcatus

Taxon classificationFungiEntomophthoralesAncylistaceae

B. Huang & Y. Nie sp. nov.

D2B22C5B-61B4-5CC0-97A7-9C2ACFFD9A4A

831599

Facesoffungi: FoF 08142

Figure 2

##### Typification.

China, Jiangsu: Nanjing, Laoshan National Forest Park, 32°6'7"N, 118°36'17"E, from plant debris, 1 Dec 2018, *Y. Nie and Y. Gao* (holotype HMAS 248359, ex-holotype culture CGMCC 3.15889 = RCEF 6551, GenBank: nucLSU = MN061285; TEF1 = MN061482; mtSSU = MN061288).

##### Etymology.

*bifurcatus* (Lat.), referring to secondary conidiophores often branched at the tip to form two short stipes, each bearing a secondary conidium.

##### Ecology and distribution.

Plant debris in Jiangsu Province, China.

##### Description.

Colonies on PDA at 21 °C for 3 d, opaque, white, reaching ca. 2 mm in diameter, with many small colonies around the periphery due to discharged conidia. Mycelia colourless, 8–11 μm wide, rarely branched and non-septate when young, often septate and distended to a width of 10–27 μm after 5 d. Primary conidiophores arising from the hyphal segments, colourless, 38–254 × 7.5–12 μm, unbranched and producing a single globose conidium, without widening upwards near the tip. Primary conidia forcibly discharged, globose to subglobose, 2–40 × 2–33 μm, with a papilla more or less tapering and pointed, 7–11 μm wide at the base, 3–12 μm long. Secondary conidiophores arising from the primary conidia, often branched almost at the tip, forming two short stipes each bearing a secondary conidium. Secondary conidia similar to, but smaller than the primary ones, mostly forcibly discharged, occasionally falling off and leaving a relic of the secondary conidiophores. On 2 % water agar, microconidia produced readily, globose to ellipsoidal, 7–12 × 6–9 μm. Zygospores homothallic, usually formed between adjacent segments of the same hypha after an incubation of 5–7 d at 21 °C on PDA, smooth, mostly globose, 25–40 μm in diameter, with a 1.5–3 μm thick wall.

##### Notes.

*Conidiobolus bifurcatus* sp. nov. is characterised by its secondary conidiophores, which are often bifurcated near the tip and bear a secondary conidium on each stipe. Morphologically, it is allied to *Conidiobolus mycophilus* Srin. & Thirum., which has smaller primary conidia (Srinivasan and Thirumalachar 1965). It appears to be similar to *C. incongruus* Drechsler and *C. mycophagus* Srin. & Thirum. in the size of primary conidia and zygospores and the formation of microconidia, but different in its longer primary conidiophores (Drechsler 1960; Srinivasan and Thirumalachar 1965). However, it is distantly related to these two species in the molecular phylogenetic tree. Instead, it is phylogenetically closely related to *C. brefeldianus* Couch (Figure 1: MP 71/ML 89/BI 1.00), but morphologically distinct by its larger primary conidia and zygospores (Couch 1939).

**Figure 2.**
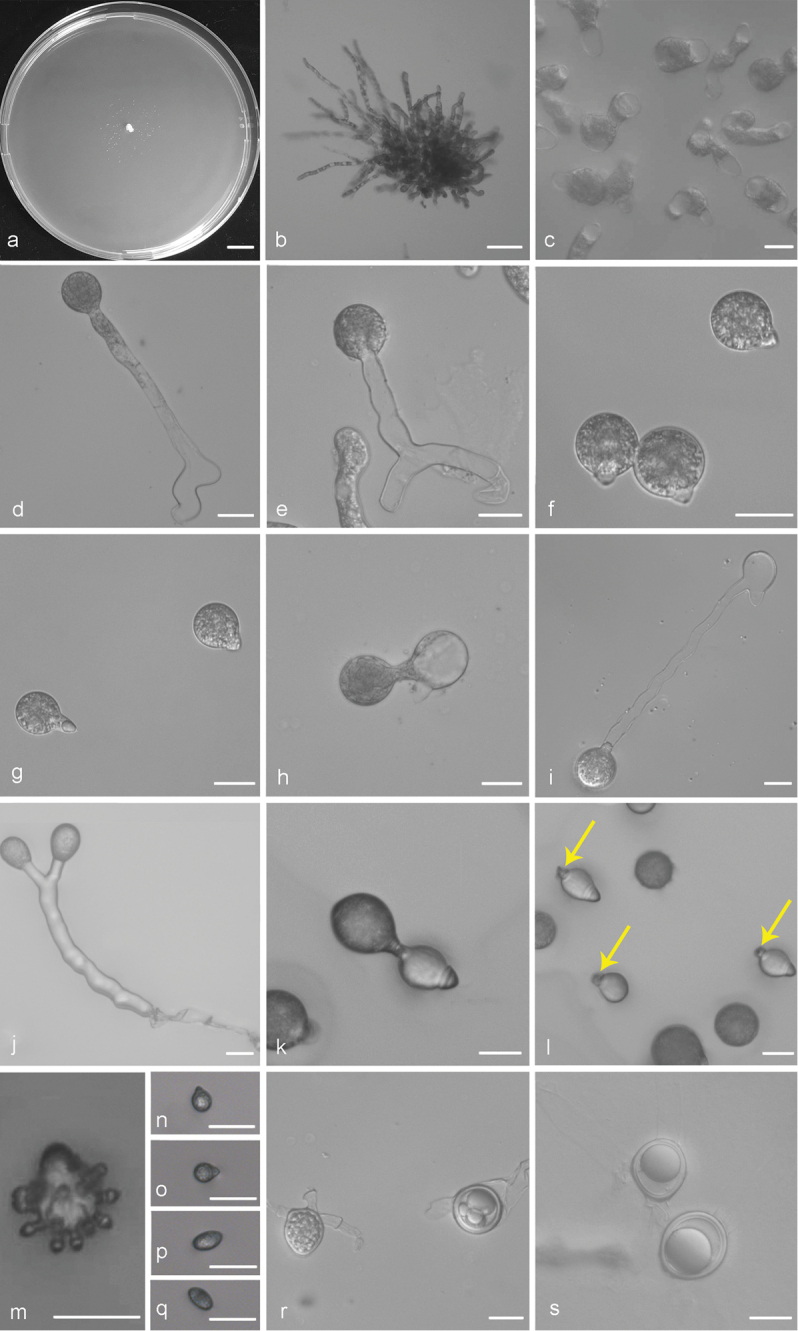
*Conidiobolus bifurcatus* sp. nov. **a** Colony on PDA after 3 d at 21 °C **b** mycelium **c** septate mycelium and distended segments **d, e** primary conidiophores bearing primary conidia **f, g** primary conidia **h, i** a single secondary conidium produced from primary conidia **j** two secondary conidia arising from a branched conidiophore **k** secondary conidia falling from primary conidia **l** the relic of secondary conidiophores on secondary conidia (arrows) **m** microconidia arising from a conidium **n, o** globose microconidia **p, q** ellipsoidal microconidia **r** zygospores formed between adjacent segments of the same hypha **s** zygospores. Scale bars: 10 mm (**a**); 100 μm (**b**); 20 μm (**c–s**).

#### Conidiobolus taihushanensis

Taxon classificationFungiEntomophthoralesAncylistaceae

B. Huang & Y. Nie sp. nov.

B3CB22D2-BA2D-59C3-BE80-F81B08F9270D

835124

Facesoffungi: FoF 08143

Figure 3

##### Typification.

China, Anhui: Ma’anshan City, Hanshan County, Taihushan National Forest Park, 31°30'53"N, 118°2'49"E, from plant detritus, 12 Jan 2019, *Y. Nie and Y. Cai* (holotype HMAS 248724, ex-holotype culture CGMCC 3.16016 = RCEF 6559, GenBank: nucLSU = MT250086; TEF1 = MT274290; mtSSU = MT250088).

##### Etymology.

*taihushanensis* (Lat.), referring to the region where the fungus was isolated.

##### Ecology and distribution.

Plant debris in Anhui Province, China.

##### Description.

Colonies on PDA at 21 °C after 3 d, white, reaching ca. 11–14 mm in diameter. Mycelia colourless, straight and unbranched when young, 8.5–12 μm wide; distended and non-contiguously segmented when old, 10–20 μm wide. Primary conidiophores arising from the older mycelia without an upward widening near the tip, colourless, 44–180 × 7–13 μm, usually unbranched and often producing a single globose primary conidium, at the initial growth stage 2–5 short branches bearing a primary conidium each. Primary conidia forcibly discharged, mostly subglobose, 27–42 × 19–32 μm, with tapering and pointed papilla, 4–10 × 8–12 μm. Secondary conidia arising from primary conidia, similar to, but smaller than the primary ones, forcibly discharged. On 2% water agar, microconidia not observed. Zygospores usually formed between adjacent segments of the same hypha after 5 d, 34–48 × 23–40 μm, with a 2–4 μm thick wall, ellipsoid and rich in content when young, smooth, mostly globose, subglobose to ovate when mature.

##### Notes.

*Conidiobolus taihushanensis* sp. nov. is morphologically highly distinct with its straight apical mycelia and the production of 2–5 conidia from the hyphal body. *Conidiobolus taihushanensis* sp. nov. is similar to *C. polytocus* Drechsler in the structure of several short branches at the top of conidiophores, but the latter is distinguished by smaller primary conidia (12–25 × 14–29 μm) and slightly curved mycelia (Drechsler 1955c). *Conidiobolus taihushanensis* sp. nov. is related to *C. margaritatus* B. Huang, Humber & K.T. Hodge and *C. megalotocus* Drechsler by the size of primary conidia, but *C. margaritatus* forms a chain of undischarged repetitional conidia (Huang et al. 2007) and *C. megalotocus* lacks zygospores (Drechsler 1956). Phylogenetically, *C. taihushanensis* sp. nov. is closely related to *C. megalotocus* (Figure 1: MP 73/BI 1.00) and distantly related to *C. polytocus*, though no molecular data are available for *C. margaritatus*. Phylogenetically, *C. taihushanensis* sp. nov. is also closely related to *C. lamprauges* Drechsler and *C. coronatus* Batko, but it differs from *C. lamprauges* by larger primary conidia (27–42 × 19–32 μm vs. 12.5–20 × 15–22 μm) and from *C. coronatus* by the absence of villose resting spores (Drechsler 1953a).

**Figure 3.**
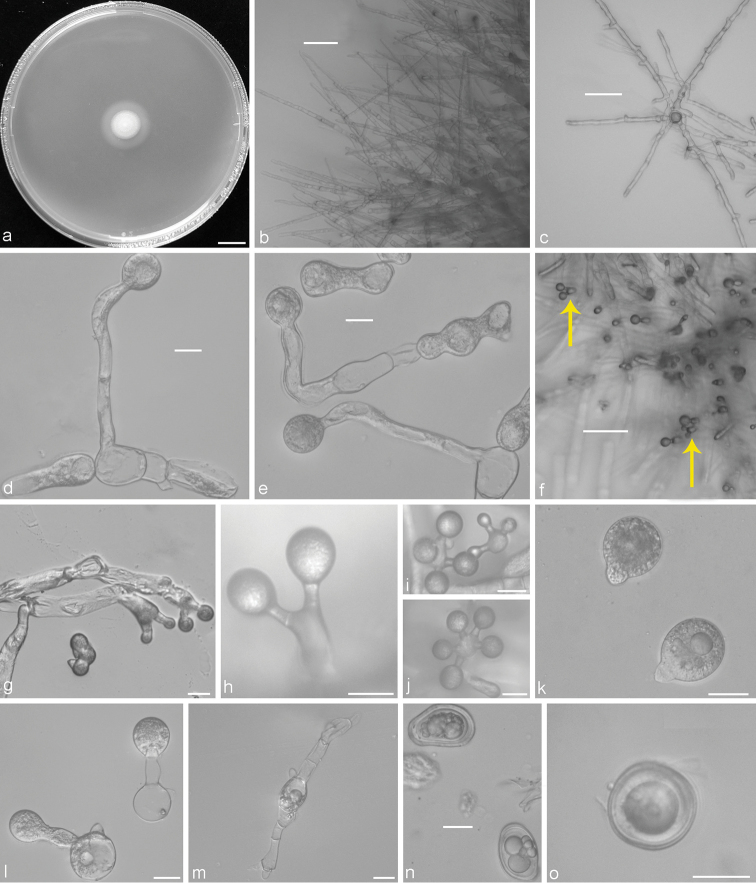
*Conidiobolus taihushanensis* sp. nov. **a** colony on PDA after 3 d at 21 °C **b** mycelia unbranched at the colony edge **c** young mycelia **d, e** primary conidiophores arising from mycelia segments **f** two branches germinated from hyphal bodies and each bearing a primary conidium (arrows) **g– j** two, three, four or five branches germinated from hyphal bodies and each bearing a primary conidium **k** globose to subglobose primary conidia **l** secondary conidia arising from primary conidia **m** zygospores formed between adjacent segments of the same hypha **n** young zygospores **o** mature zygospores. Scale bars: 10 mm(**a**); 100 μm (**b, c, f**); 20 μm (**d, e, g–o**).

#### Conidiobolus variabilis

Taxon classificationFungiEntomophthoralesAncylistaceae

B. Huang & Y. Nie sp. nov.

68BD96CB-9D9F-5C96-AB12-2C8B36A9DC86

835125

Facesoffungi: FoF 08144

Figure 4

##### Typification.

China, Anhui: Ma’anshan City, Hexian County, Jilongshan National Forest Park, 31°48'1"N, 118°12'19"E, from plant debris, 23 Dec 2017, *Y. Nie* (holotype HMAS 248723, ex-holotype culture CGMCC 3.16015 (= RCEF 6540), GenBank: nucLSU = MT250085; TEF1 = MT274289; mtSSU = MT250087).

##### Etymology.

*variabilis* (Lat.), referring to producing various shapes of primary conidia.

##### Ecology and distribution.

Plant debris from Anhui Province, China.

##### Description.

Colonies on PDA at 21 °C after 3 d white, reaching ca. 41–48 mm in diameter. Mycelia colourless, 6–11 μm wide, rarely branched at the colony edge. Primary conidiophores unbranched and producing a single globose conidium, colourless, 60–200 × 9–15 μm, without an upward widening near the tip. Primary conidia forcibly discharged, globose, subglobose, pyriform to oboviod, 31–55 × 25–40 μm, with tapering and pointed papilla, 3.5–9 × 8–13 μm. Secondary conidia arising from primary conidia, similar to, but smaller than primary ones, forcibly discharged. On 2% water agar, microconidia rarely observed, globose, subglobose to ellipsoidal, 10–12 × 9–14 μm. Resting spores not observed.

##### Notes.

Considering the large size of primary conidia, *Conidiobolus variabilis* sp. nov. is allied to *C. coronatus* (Cost.) Batko (14.5–38.5 × 17–48.5 μm), *C. macrosporus* Srin. & Thirum. (38–45 × 48–54 μm) and *C. utriculosus* Brefeld (25–35 × 37.5–51 μm). It is distinguished from *C. coronatus* by its various shapes of primary conidia and the absence of villose spores. It differs from *C. macrosporus* by its longer primary conidiophores and the absence of resting spores (Batko 1964, Srinivasan and Thirumalachar 1967). It is differentiated from *C. utriculosus* by the shapes of primary conidia and the absence of zygospores. Phylogenetically, *C. variabilis* sp. nov. is basal in clade I and distantly related to *C. coronatus* and *C. macrosporus*.

**Figure 4.**
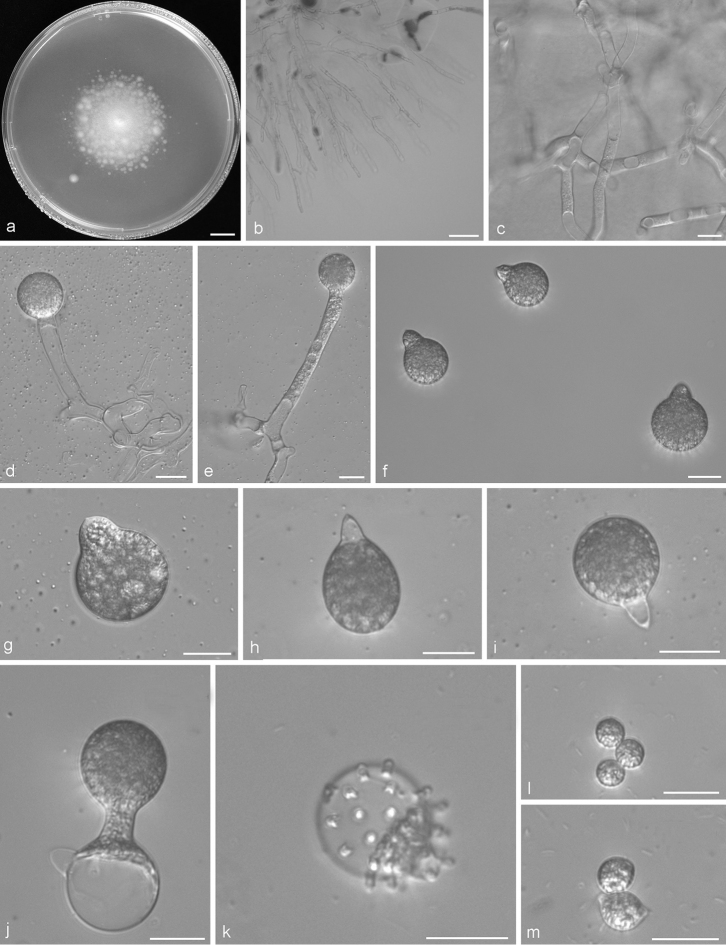
*Conidiobolus variabilis* sp. nov. **a** Colony on PDA after 3 d at 21 °C **b** mycelia rarely branched at the colony edge **c** mycelia **d, e** primary conidiophores bearing primary conidia **f–i** primary conidia with different shapes **j** secondary conidia arising from primary conidia **k** microconidia arising from conidia **l** globose microconidia **m** ellipsoidal microconidia. Scale bars: 10 mm (**a**); 100 μm (**b**); 20 μm (**c–m**).

## Discussion

The genus *Conidiobolus* has recently been divided into four lineages and one of them was treated as *Conidiobolus* sensu stricto on the basis of a synapomorph, namely microspores (Nie et al. 2020). The three new species *C. bifurcatus* sp. nov., *C. taihushanensis* sp. nov. and *C. variabilis* sp. nov. are located in the clade of *Conidiobolus* s.s. (Fig. 1). *Conidiobolus taihushanensis* sp. nov. was paraphyletic to *C. megalotocus* Drechsler, *C. lamprauges* Drechsler and *C. coronatus* (Cost.) Batko with a robust support of BI posterior probability of 1.00. *Conidiobolus bifurcatus* sp. nov. was a sister group to *C. brefeldianus*, which was supported by all three inferring methods (MP 71/ML 89/BI 1.00). *Conidiobolus variabilis* sp. nov. was basal in clade I with a relatively high confidence (MP 97/ML 96/BI 1.00). *Conidiobolus bifurcatus* sp. nov. and *C. variabilis* sp. nov. morphologically produce microspores. However, *C. taihushanensis* sp. nov. lacks this synapomorph. Besides *C. taihushanensis* sp. nov., four other species in the *Conidiobolus* s.s., i.e. *C. dabieshanensis* Y. Nie & B. Huang, *C. iuxtagenitus* S.D. Waters & Callaghan, *C. lamprauges* and *C. parvus* Drechsler were not reported to produce microspores either. This may be due to the need for particular conditions, such as growth temperature and nutritional supply. For example, the microspores of *C. khandalensis* Srin. & Thirum. were only observed on 2% water-agar at 16 °C (Nie et al. 2020).

Except microspores, species of the *Conidiobolus* s.s. clade are morphologically diverse, particularly the secondary conidia. For instance, *C. iuxtagenitus* produces single fusiform discharged secondary conidia (Waters and Callaghan 1989) and *C. margaritatus* forms a necklace-like chain of up to seven undischarged conidia (Huang et al. 2007). Although these special characteristics provide good identification, most members of this lineage are difficult to distinguish phenotypically. Sequence data of nucLSU and TEF1 have provided a better understanding of species circumscription or inter- and intraspecific variations (Nie et al. 2012). In this study, morphology and molecular data support *C. bifurcatus* sp. nov., *C. taihushanensis* sp. nov. and *C. variabilis* sp. nov. as new species in the *Conidiobolus* s.s. clade. Although the microspores of *C. taihushanensis* sp. nov. were not observed, its straight apical mycelium and the production of 2–5 conidia from the hyphal body make it easily distinguishable from other species of *Conidiobolus* s.s.

With the proposal of the three new species herein, 17 species are currently accepted in the genus *Conidiobolus* s.s. and only five were found distributed in China (King 1976a, b, 1977, Wang et al. 2010a, b, Nie et al. 2017, 2020). For updating, the key to all these 17 species are provided as follows.

### Key to the species of *Conidiobolus* s.s.

**Table:** 

1	Villose resting spores produced	***Conidiobolus coronatus***
–	Villose resting spores not produced	**2**
2	Microspores produced	**3**
–	Microspores not observed	**4**
3	Two types of sexual reproduction, zygospores formed in axial alignment with one or both conjugating segments	**5**
–	One type of sexual reproduction, zygospores formed in one of the conjugating segments	**6**
5	Primary conidia larger, up to 51 μm	***C. utriculosus***
–	Primary conidia smaller, less than 36 μm	***C. brefeldianus***
6	2–4 branches germinated at the top of primary conidiophores	**7**
–	Unbranched at the top of conidiophores	**8**
7	Only 2 primary conidia arising from 2 branches, larger, up to 44 μm	***C. megalotocus***
–	2–4 primary conidia arising from 2–4 branches, smaller, less than 29 μm	***C. polytocus***
8	Secondary conidiophores branched	**9**
–	Secondary conidiophores unbranched	**10**
9	Secondary conidiophores branched almost at the tip, primary conidia larger, up to 40 μm	***C. bifurcatus* sp. nov.**
–	Secondary conidiophores branched at the tip or base, primary conidia smaller, less than 30 μm	***C. mycophilus***
10	Primary conidia larger, up to 55 μm	**11**
–	Primary conidia smaller, maximum not over 42 μm	**12**
11	Primary conidia globose to pyriform, zygospores globose, 26–40 μm	***C. macrosporus***
–	Primary conidia globose, subglobose, pyriform to oboviod, zygospores not observed	***C. variabilis* sp. nov.**
12	Primary conidia smaller, less than 21 μm	***C. khandalensis***
–	Primary conidia larger, more than 33 μm	**13**
13	Two types of resting spores produced: zygospores or chlamydospores	***C. humicolus***
–	One type of resting spores produced	**14**
14	Only chlamydospores produced	***C. firmipilleus***
–	Only zygospores produced	**15**
15	Primary conidiophores shorter, less than 80 μm	***C. gonimodes***
–	Primary conidiophores longer, more than 130 μm	**16**
16	Zygospores globose or elongate, larger, 15–40 × 18–45 μm	***C. incongruus***
–	Zygospores globose, smaller, 30–36 μm	***C. mycophagus***
4	Fusiform secondary conidia produced, each zygospore in a position separated by a short, but relatively constant distance from a lateral conjugation outgrowth or beak	***C. iuxtagenitus***
–	Fusiform secondary conidia not produced, each zygospore in a position not separated by a short, but relatively constant distance from a lateral conjugation outgrowth or beak	**17**
17	A chain of up to seven undischarged repetitional conidia produced	***C. margaritatus***
–	No chains of undischarged repetitional conidia produced	**18**
18	Primary conidiophores produced from cushion mycelia	***C. lichenicolus***
–	Primary conidiophores not produced from cushion mycelia	**19**
19	Apical mycelia straight, 2–5 conidia arising from hyphal body, no chlamydospores, zygospores produced	***C. taihushanensis* sp. nov.**
–	Apical mycelia slightly curved, unbranched at the top of conidiophore, chlamydospores produced, no zygospores	***C. dabieshanensis***

## Supplementary Material

XML Treatment for Conidiobolus bifurcatus

XML Treatment for Conidiobolus taihushanensis

XML Treatment for Conidiobolus variabilis

## References

[B1] Bałazy S Wiśniewski J Kaczmarek S. (1987) Some noteworthy fungi occurring on mites. Bulletin of the Polish Academy of Sciences, Biological Sciences 35: 199–224.

[B2] Balazy S (1993) Entomophthorales Flora of Poland (Flora Polska), Fungi (Mycota) 24:1–356. Polish Academy of Sciences, W. Szafer Institute of Botany, Kraków, Poland.

[B3] Batko A (1964) Notes on entomophthoraceous fungi in Poland Entomophaga. Mémoires hors série. 2: 129–131.

[B4] Ben-Ze’ev IS Kenneth RG (1982) Features-criteria of taxonomic value in the *Entomophthorales*: I. A revision of the *Batkoan* classification. Mycotaxon 14: 393–455.

[B5] Brefeld O (1884) *Conidiobolus utriculosus* und *minor*. Untersuchungen aus der Gesammtgebiete der Mykologie 6(2): 35–78.

[B6] Callaghan AA Waters SD Manning RJ (2000) Alternative repetitional conidia in *Conidiobolus adiaeretus*: development and germination. Mycological Research 104: 1270–1275. 10.1017/S0953756200003063

[B7] Chen MJ Huang B (2018) *Conidiobolus antarcticus*, a synonym of *C. osmodes*. Mycotaxon 133(4): 635–641. 10.5248/133.635

[B8] Couch JN (1939) A new *Conidiobolus* with sexual reproduction. American Journal of Botany 26: 119–130. 10.1002/j.1537-2197.1939.tb12878.x

[B9] Drechsler C (1952) Widespread distribution of *Delacroixia coronata* and other saprophytic *Entomophthoraceae* in plant detritus. Science 115: 575–576. 10.1126/science.115.2995.575 17759184

[B10] Drechsler C (1953a) Three new species of *Conidiobolus* isolated from leaf mold. Journal of the Washington Academy of Science 43(2): 29–34.

[B11] Drechsler C (1953b) Two new species of *Conidiobolus* occurring in leaf mold. American Journal of Botany 40(3): 104–115. 10.1002/j.1537-2197.1953.tb06458.x

[B12] Drechsler C (1954) Two species of *Conidiobolus* with minutely ridged zygospores. American Journal of Botany 41: 567–575. 10.1002/j.1537-2197.1954.tb14380.x

[B13] Drechsler C (1955a) A small *Conidiobolus* with globose and with elongated secondary conidia. Journal of Washington Academy of Sciences 45(4): 114–117.

[B14] Drechsler C (1955b) Three new species of *Conidiobolus* isolated from decaying plant detritus. American Journal of Botany 42(5): 437–443. 10.1002/j.1537-2197.1955.tb11144.x

[B15] Drechsler C (1955c) Two new species of *Conidiobolus* that produce microconidia. American Journal of Botany 42(9): 793–802. 10.1002/j.1537-2197.1955.tb10424.x

[B16] Drechsler C (1956) Two new species of *Conidiobolus*. American Journal of Botany 43(10): 778–787. 10.1002/j.1537-2197.1956.tb11168.x

[B17] Drechsler C (1957a) Two small species of *Conidiobolus* forming lateral zygospores. Bulletin of the Torrey Botanical Club 84(4): 268–280. 10.2307/2482673

[B18] Drechsler C (1957b) A new species of *Conidiobolus* with distended conidiophores. Sydowia Annales Mycologici 9: 189–192.

[B19] Drechsler C (1957c) Two medium-sized species of *Conidiobolus* occurring in Colorado. Journal of Washington Academy of Sciences 47: 309–315.

[B20] Drechsler C (1960) Two new species of *Conidiobolus* found in plant detritus. American Journal of Botany 47: 368–377. 10.1002/j.1537-2197.1960.tb07138.x

[B21] Drechsler C (1961) Two species of *Conidiobolus* often forming zygospores adjacent to antheridium-like distentions. Mycologia 53(3): 278–303. 10.2307/3756275

[B22] Drechsler C (1962) A small *Conidiobolus* with resting spores that germinate like zygospores. Bulletin of the Torrey Botanical Club 89(4): 233–240. 10.2307/2483199

[B23] Drechsler C (1965) A robust *Conidiobolus* with zygospores containing granular parietal protoplasm. Mycologia 57(6): 913–926. 10.2307/3756891

[B24] Felsenstein J (1985) Confidence limits on the bootstrap: an approach using the bootstrap. Evolution 38: 783–791. 10.1111/j.1558-5646.1985.tb00420.x 28561359

[B25] Gryganskyi AP Humber RA Smith ME Miadlikovska J Wu S Voigt K Walther G Anishchenko IM Vilgalys R (2012) Molecular phylogeny of the *Entomophthoromycota*. Molecular Phylogenetics and Evolution 65: 682–694. 10.1016/j.ympev.2012.07.026 22877646

[B26] Gryganskyi AP Humber RA Smith ME Hodge K Huang B Voigt K Vilgalys R (2013) Phylogenetic lineages in *Entomophthoromycota*. Persoonia 30: 94–105. 10.3767/003158513X666330 24027349 PMC3734969

[B27] Hall TA (1999) Bioedit: a user-friendly biological sequence alignment editor and analysis program for Windows 95/98/NT. Nucleic Acids Symposium Series 41: 95–98.

[B28] Huang B Humber RA Hodge KT (2007) A new species of *Conidiobolus* from Great Smoky Mountains National Park. Mycotaxon 100: 227–233.

[B29] Humber RA (1997) Fungi: identification. In: LA Lacey (Ed.) Manual of Techniques in Insect Pathology. London, Academic Press. 10.1016/B978-012432555-5/50015-4

[B30] James TY Letcher PM Longcore JE Mozley-Standridge SE Porter D Powell MJ Griffith GW Vilgalys R (2006) A molecular phylogeny of the flagellated fungi (*Chytridiomycota*) and description of a new phylum (*Blastocladiomycota*). Mycologia 98(6): 860–871. 10.1080/15572536.2006.11832616 17486963

[B31] Jensen AB Gargas A Eilenberg J Rosendahl S (1998) Relationships of the insect-pathogenic order *Entomophthorales* (*Zygomycota*, *Fungi*) based on phylogenetic analyses of nucleus small subunit ribosomal DNA sequences (SSU rDNA). Fungal Genetics and Biology 24(3): 325–334. 10.1006/fgbi.1998.1063 9756713

[B32] King DS (1976a) Systematics of Conidiobolus (Entomophthorales) using numerical taxonomy I. Taxonomic considerations. Canadian Journal of Botany 54: 45–65. 10.1139/b76-008

[B33] King DS (1976b) Systematics of Conidiobolus (Entomophthorales) using numerical taxonomy II. Taxonomic considerations. Canadian Journal of Botany 54: 1285–1296. 10.1139/b76-141

[B34] King DS (1977) Systematics of Conidiobolus (Entomophthorales) using numerical taxonomy III. Descriptions of recognized species. Canadian Journal of Botany 55: 718–729. 10.1139/b77-086

[B35] Liu M Rombach MC Humber RA Hodge KT (2005) What’s in a name? *Aschersonia insperata*: a new pleoanamorphic fungus with characteristics of *Aschersonia* and *Hirsutella*. Mycologia 97: 249–256. 10.3852/mycologia.97.1.246 16389976

[B36] Lutzoni F Kauff F Cox CJ McLaughlin D Celio G Dentinger B Padamsee M Hibbett DS James TY Baloch E Grube M Reeb V Hofstetter V Schoch C Arnold AE Miadlikowska J Spatafora J Johnson D Hambleton S Crockett M Schoemaker R Sun GH Lücking R Lumbsch HT O’Donnell K Binder M Diederich P Ertz D Gueidan C Hall B Hansen K Harris RC Hosaka K Lim YW Liu Y Matheny B Nishida H Pfister D Rogers J Rossman A Schmitt I Sipman H Stone J Sugiyama J Yahr R Vilgalys R (2004) Where are we in assembling the Fungal Tree of Life, classifying the fungi and understanding the evolution of their subcellular traits? American Journal of Botany 91(10): 1446–1480. 10.3732/ajb.91.10.1446 21652303

[B37] Nie Y Yu CZ Liu XY Huang B (2012) A new species of Conidiobolus (Ancylistaceae) from Anhui, China. Mycotaxon 120: 427–435. 10.5248/120.427

[B38] Nie Y Tang XX Liu XY Huang B (2016) *Conidiobolus stilbeus*, a new species with mycelial strand and two types of primary conidiophores. Mycosphere 7(6): 801–809. 10.5943/mycosphere/7/6/11

[B39] Nie Y Tang XX Liu XY Huang B (2017) A new species of *Conidiobolus* with chlamydospores from Dabie Mountains, eastern China. Mycosphere 8(7): 809–816. 10.5943/mycosphere/8/7/1

[B40] Nie Y Qin L Yu DS Liu XY Huang B (2018) Two new species of *Conidiobolus* occurring in Anhui, China. Mycological Progress 17(10): 1203–1211. 10.1007/s11557-018-1436-z

[B41] Nie Y Yu DS Wang CF Liu XY Huang B (2020) A taxonomic revision of the genus *Conidiobolus* (*Ancylistaceae*, *Entomophthorales*): four clades including three new genera. Mycokeys 66: 55–81. 10.3897/mycokeys.66.46575 32273794 PMC7136305

[B42] Posada D Crandall KA (1998) MODELTEST: testing the model of DNA substitution. Bioinformatics 14: 817–818. 10.1093/bioinformatics/14.9.817 9918953

[B43] Rambaut A (2012) FigTree version 1.4.0. Available at http://tree.bio.ed.ac.uk/software/figtree/

[B44] Ronquist F Huelsenbeck JP (2003) MRBAYES 3: Bayesian phylogenetic inference under mixed models. Bioinformatics 19: 1572–1574. 10.1093/bioinformatics/btg180 12912839

[B45] Silvestro D Michalak I (2012) raxmlGUI: a graphical front-end for RAxML. Organisms Diversity & Evolution 12: 335–337. 10.1007/s13127-011-0056-0.

[B46] Srinivasan MC Thirumalachar MJ (1961) Studies on species of *Conidiobolus* from India-I. Sydowia, Annales Mycologici 15: 237–241.

[B47] Srinivasan MC Thirumalachar MJ (1962a) Studies on species of *Conidiobolus* from India-II. Sydowia, Annales Mycologici 16: 60–66.

[B48] Srinivasan MC Thirumalachar MJ (1962b) Studies on species of *Conidiobolus* from India-III. Mycologia 54(6): 685–693. 10.2307/3756504

[B49] Srinivasan MC Thirumalachar MJ (1965) Studies on species of *Conidiobolus* from India-IV. Sydowia 19: 86–91.

[B50] Srinivasan MC Thirumalachar MJ (1967) Evaluation of taxonomic characters in the genus *Conidiobolus* with key to known species. Mycologia 59: 698–713. 10.2307/3757098

[B51] Srinivasan MC Thirumalachar MJ (1968a) Studies on species of *Conidiobolus* from India-V. Mycopathologica et Mycologia Applicata 36: 341–346. 10.1007/BF02050380

[B52] Srinivasan MC Thirumalachar MJ (1968b) Two new species of *Conidiobolus* from India. Journal of the Mitchell Society 84: 211–212.

[B53] Stamatakis A (2006) RAxML-VI-HPC: maximum likelihoodbased phylogenetic analyses with thousands of taxa and mixed models. Bioinformatics 22: 2688–2690. 10.1093/bioinformatics/btl446 16928733

[B54] Swofford DL (2002) PAUP*: Phylogenetic analysis using parsimony (*and other methods), Version 4.0b10. Sinauer Associates, Sunderland.

[B55] Thompson JD Gibson TJ Plewniak F (1997) The Clustal-X windows interface: flexible strategies for multiple sequence alignment aided by quality analysis tools. Nucleic Acids Research 63: 215–228. 10.1093/nar/25.24.4876 PMC147148 9396791

[B56] Vaidya G Lohman DJ Meier R (2011) SequenceMatrix: concatenation software for the fast assembly of multi-gene datasets with character set and codon information. Cladistics 27(2): 171–180. 10.1111/j.1096-0031.2010.00329.x 34875773

[B57] Vilgalys R Hester M (1990) Rapid genetic identification and mapping of enzymatically amplified ribosomal DNA from several *Cryptococcus* species. Journal of Bacteriology 172: 4238–4246. 10.1128/jb.172.8.4238-4246.1990 2376561 PMC213247

[B58] Voigt K Cigelnik E O’donnell K (1999) Phylogeny and PCR identification of clinically important zygomycetes based on Nuclear Ribosomal-DNA Sequence Data. Journal of Clinical Microbiology 37(12): 3957–3964. 10.1128/JCM.37.12.3957-3964.1999 10565914 PMC85855

[B59] Waingankar VM Singh SK Srinivasan MC (2008) A new thermophilic species of *Conidiobolus* from India. Mycopathologia 165: 173–177. 10.1007/s11046-007-9088-6. 18266074

[B60] Wang CF Li KP Huang B (2010a) A new record to China — *Conidiobolus iuxtagenitus*. Journal of Fungal Research 8: 12–14.

[B61] Wang CF Li KP Liu YJ Li ZZ Huang B (2010b) Three new Chinese records of *Conidiobolus*. Mycosystema 29: 595–599.

[B62] Watanabe M Lee K Goto K Kumagai S Sugita-Konishi Y Hara-Kudo Y (2010) Rapid and effective DNA extraction method with bead grinding for a large amount of fungal DNA. Journal of Food Protection 73(6): 1077–1084. 10.4315/0362-028X-73.6.1077 20537263

[B63] Waters SD Callaghan AA (1989) *Conidiobolus iuxtagenitus*, a new species with discharge delongate repetitional conidia and conjugation tubes. Mycological Research 93: 223–226. 10.1016/S0953-7562(89)80121-2

[B64] Zollera S Scheideggera C Sperisena C (1999) PCR primers for the amplification of mitochondrial small subunit ribosomal DNA of lichen-forming ascomycetes. The Lichenologist 31(5): 511–516. 10.1006/lich.1999.0220

